# Malpractice in a Case of Midwifery

**Published:** 1845-06

**Authors:** 


*Malpractice in a Case of Midwifery.*—Another of those cases of gross ignorance or extreme carelessness in a medical attendant, which seem to be either much more common in England than in this country, or more readily reach the public ear there, has lately occurred in Norfolk. At the coroner's inquest, it was proved that Mr. Gatches, when in attendance upon Mrs. Lovett, in labor with her tenth child, attempted to remove the after-birth in an hour or two after delivery. Upon making efforts for this purpose, he withdrew a substance which the midwife and nurse described as a round, brown, firm heavy substance, with a string attached to it, which Mr. Gaches took away with him. The woman died in half an hour. A *post-mortem* examination was made, and the surgeon making it testified that the womb and a large portion fo the intestines had been removed, which he had no doubt was the cause of death. The coroner's jury returned a verdict of "manslaughter" against Mr. Gaches, who said the accident was altogether an error of judgment. He was given in custody of two policemen, who gave him time and liberty to find bail, which he improved by escaping before the next morning. Mr. G. had a diploma from the Apothecaries' Company, but was not a member of the College of Surgeons.—*Bost. Med. and Surg. Jour.*

